# Long‐COVID‐19 Symptoms are associated with Abnormal Cognitive Testing and Mild Cognitive Impairment Compared to Controls

**DOI:** 10.1002/alz70857_103528

**Published:** 2025-12-25

**Authors:** Jennifer A. Frontera, Arjun V. Masurkar, Alok Vedvyas, Rebecca A Betensky, Allal Boutajangout, Manisha Bohara, Joshua Chodosh, Salma Hammam, Li Jiang, Jon Links, Karyn Marsh, Sujata Thawani, Daria Vasilchenko, Amin Yakubov, Yulin Ge, Thomas Wisniewski

**Affiliations:** ^1^ NYU Grossman School of Medicine, New York, NY, USA; ^2^ Alzheimer's Disease Research Center, New York University Langone Health, New York, NY, USA; ^3^ Center for Cognitive Neurology, New York University Langone Health, New York, NY, USA; ^4^ NYU Alzheimer's Disease Research Center, New York, NY, USA; ^5^ NYU College of Global Public Health, New York, NY, USA; ^6^ Center for Cognitive Neurology, New York, NY, USA; ^7^ New York University Grossman School of Medicine, New York, NY, USA; ^8^ NYU Langone Hospitals, New York, NY, USA; ^9^ Vilcek Institute of Biomedical Science, New York University School of Medicine, New York, NY, USA

## Abstract

**Background:**

There is a paucity of data comparing neuropsychiatric testing in patients with post‐acute COVID‐19 symptoms (“long‐COVID”) to COVID‐19 patients without long‐term symptoms and to COVID‐19 negative controls.

**Method:**

We conducted a cross‐sectional study of patients with and without prior laboratory‐confirmed COVID‐19 (COV+ vs COV‐), who had no previous history of dementia/cognitive impairment. Long‐COVID was defined as subjective symptoms (WHO symptom questionnaire) lasting >1 month after index SARS‐CoV‐2 infection. All patients underwent UDS3 neuropsychiatric testing and received a formal cognitive diagnosis (with respective etiology following NACC criteria) by a consensus group of physicians blinded to their long‐COVID status as: normal, mild‐cognitive impairment (MCI), or dementia. Cognitive test scores, CDR, and physician diagnoses were compared between patients with and without long‐COVID using Fisher's exact and Mann‐Whitney U tests and multivariable logistic regression models were constructed to adjust for differences in age, sex, time from initial COVID diagnosis and number of COVID‐19 infections.

**Result:**

We enrolled 279 patients: *N* = 51 (18%) COV‐, *N* = 228 (82%) COV+, *N* = 122 (44%) with long‐COVID, median age 70 years (IQR 63‐76), and 62% female. Compared to COV‐ subjects and COV+ subjects without long‐COVID, participants with long‐COVID had significantly worse cognitive test results (≥1 cognitive battery abnormal in 64% with long‐COVID compared to 46% without, *p* = 0.003), as well as worse global CDR and CDR sum of boxes scores (26% CDR>0 among long‐COVID vs. 6% without long‐COVID, *p* <0.001). Significantly more long‐COVID patients were diagnosed with MCI or dementia (25% vs. 6% without long‐COVID, *p* <0.001), including higher proportions with MCI related to Alzheimer's disease (10% vs. 3%, *p* = 0.038), and MCI related to psychiatric diagnoses (11% vs. 1%, *p* <0.001). After adjusting for sex, age, time from initial COVID diagnosis and number of COVID‐19 infections, long‐COVID was associated with significantly higher odds of an MCI diagnosis (aOR 3.5, 95% CI 1.2‐10.6, *p* = 0.025), and specifically MCI due to Alzheimer's (aOR 4.4, 95% CI 1.1‐18.3, *p* = 0.041), but not MCI due to psychiatric disease.

**Conclusion:**

Long‐COVID patients had significantly worse cognitive test scores and higher rates of MCI (Alzheimer's type) diagnoses compared to both COV‐ and COV+ subjects without long‐COVID symptoms.

